# Effects of Changes in Pectin Constitution on Optical Properties and Firmness of Peach Flesh during Storage

**DOI:** 10.3390/foods13193042

**Published:** 2024-09-25

**Authors:** Xiao Chen, Chen Ma, Hongju He, Kang Tu, Weijie Lan, Leiqing Pan

**Affiliations:** 1 College of Food Science and Technology, Nanjing Agricultural University, No. 1 Weigang Road, Nanjing 210095, China; xiaochen@njau.edu.cn (X.C.); kangtu@njau.edu.cn (K.T.); weijie.lan@njau.edu.cn (W.L.); 2 Xuzhou Institute of Agricultural Sciences in Jiangsu Xuhuai Area, Jiangsu Academy of Agricultural Sciences, Xuzhou 221131, China; 20201003@jaas.ac.cn; 3 College of Light Industry and Food Engineering, Nanjing Forestry University, Nanjing 210037, China; hongju.he@hist.edu.cn

**Keywords:** peach flesh, firmness, pectin constitution, optical properties, partial least squares (PLS)

## Abstract

Understanding the fundamental light-sample interaction process is a crucial step toward the development of vibrational spectroscopy to determine fruit texture (i.e., firmness). This study aimed to investigate the effect of pectin constitution, including total pectin, water-soluble pectin, protopectin contents, and protopectin index (*PI*), on the optical properties and firmness of ‘Baifeng’ and ‘Xiahui 8’ peach flesh at the different softening degrees during postharvest storage of 6 days at 20 °C. The firmness of ‘Baifeng’ and ‘Xiahui 8’ peaches significantly (*p* < 0.05) changed with a decreasing rate from 90.3% to 92.2%. Peach firmness of these two cultivars correlated well with *PI* contents (*r* > 0.912) and showed good internal correlations with optical scattering properties. The light absorption coefficient (*μ_a_*) and reduced scattering coefficient (*μ’_s_*) at 600–1600 nm were measured using a single integrating sphere system combined with an inversion algorithm. This relationship of *μ_a_* and *μ’_s_* with peach firmness and pectin constitution was first analyzed. Notably, the specific *μ’_s_* at 660 nm, 950 nm, 1203 nm, and 1453 nm showed a satisfactory prediction of peach firmness and *PI* of ‘Xiahui 8’ (*R*^2^ ≥ 0.926) and ‘Baifeng’ peaches (*R*^2^ ≥ 0.764), respectively. Furthermore, the prediction models were established based on partial least squares regression coupled with optical properties, and considerable prediction performances were obtained for tissue firmness (*R_p_*^2^ ≥ 0.863) and *PI* based on *μ’_s_* (*R_p_*^2^ ≥ 0.802). Consequently, these results further verified that the spectroscopic prediction model for peach firmness could be related to the high correlations between *PI* in tissues and their optical scattering properties. Future research interests could include the development of optical absorption and scattering sensors for rapid and efficient determination of peach firmness.

## 1. Introduction

Peach (*Prunus persica* L. Batsch), an important stone fruit, has a sweet taste and high nutritional value [1]. Owing to different textural changes during ripening, peach cultivars are classified into melting, non-melting, and stony hard types [2]. Firmness is a major internal quality factor of peach fruit, which strongly affects the purchase behaviors of consumers [3]. According to the previous works, intensive variations in fruit textures were observed at different storage conditions, and it could be strongly related to the changes in cell wall structure and constitution [4]. Particularly, pectin is a complex structural polymer mainly composed of D-gal acturonic acid that is considered the main composition of fruit cell walls and changes that alter the firmness and other mechanical properties of fruit tissue. Pectin plays a crucial role in determining the texture and firmness of plant tissues, particularly in fruits and vegetables. Pectin is a major component of the middle lamella, the layer of cell wall material that adheres adjacent cells together. Its highly hydrated and gel-like nature provides a strong, cohesive force between cells, contributing to the overall rigidity and structural integrity of plant tissues. By maintaining this cohesive strength, pectin helps to keep fruits and vegetables firm and resistant to deformation or damage [4,5]. Under natural conditions, pectin exists as insoluble pectin, soluble pectin, and pectin acid. The solubilization of pectin is one of the dominant reasons for the decrease in peach firmness during storage. While fruit is ripening, the insoluble pectin is noticed to convert into soluble form, and the pectin chain length decreases. Therefore, one of our major interests in this work is to provide deep insights into the specific relationships between peach firmness and different pectin compositions in peach tissues during postharvest storage.

Conventional determination methods of fruit firmness mainly depend on the puncture test, which is destructive, time-consuming, and not suitable for numerous characterization [6]. Moreover, the characterization of pectin contents is based on gas chromatography and mass spectrometry or high-performance liquid chromatography, which suffers from the drawbacks of cumbersome chemical wastes and the recruitment of experienced operators [7]. The infrared spectroscopic technique can be used as an efficient and non-destructive strategy to evaluate the internal chemical and structural characteristics of fruit, depending on the specific vibrations of molecular bonds, such as C-H, O-H, and N-H. It is widely used for evaluating fruit internal quality using reflection/transmission spectra, which yield internal fruit information and establish the relationship between the spectrum and fruit quality [7]. The citrus fungal disease was detected by non-destructive identification, which obtained the highest accuracy of the classification by the back-propagation neural network classifier [8]. Some studies have used spatial frequency domain imaging to obtain optical properties, which were used as variables to classify fruit according to either the class of intensity for every attribute or according to the sensory profile [9]. Currently, the applications of infrared spectroscopy to determine fruit firmness are always combined with multivariate data analysis and stoichiometry to build prediction models. The poor model robustness of the fruit firmness prediction, taking into account a large variability of the testing samples, is the major challenging work [10]. One of the main reasons could be the spectral technology based on the Lambert–Beer law only considers the absorption of light, which affects the prediction accuracy [11]. Till now, there are limited reports providing specific insight into the effect of changes in peach tissue firmness on optical absorption and scattering.

Light interaction with fruit tissues can be characterized by two optical parameters: the absorption coefficient (*μ_a_*) and reduced scattering coefficient (*μ’_s_*). The *μ_a_* coefficient can be used to describe the chemical compositions of fruit, whereas the *μ’_s_* coefficients are related to tissue microstructural and physical features. The knowledge of these optical property parameters is critical for understanding the light interaction with biological materials and the relationship between chemical components, physical structure, and optical properties independently [12,13]. The measurement of optical properties mainly includes time-resolved (TR), frequency domain (FD), spatially resolved (SR), and integrating sphere (IS) technology, among which IS is highly accurate and inexpensive [14]. Recently, optical coefficients have been successfully used to inspect fruit quality indexes. They were successfully used to evaluate the soluble solids contents and firmness for different kinds of fruit, including apples [15,16] and bananas [17]. Further, the previous work highlighted that *μ’_s_* at 675 nm were highly related to apple firmness and showed the potential correlations of the water-soluble pectin and the residual insoluble pectin [18]. Hence, the optical property measurements could provide the opportunity to explore the relationship between the optical parameters of peach tissues and their firmness and pectin component changes.

Therefore, this study aimed to investigate the effect of pectin constitution on the firmness and optical properties of peach tissues during postharvest storage and further explored the fundamental mechanism of firmness prediction using optical technology. The optical absorption and scattering properties were characterized on two peach cultivars with a large texture diversity at different softening degrees during postharvest storage based on a single integrating sphere (SIS) system from 600 nm to 1650 nm, as well as their firmness and pectin contents of tissues, with the specific objectives to do the following:

(i) explore the internal variation and relationship between optical properties and the firmness and pectin compositions (water-soluble pectin, protopectin, and the protopectin index) of peach flesh tissues;
(ii) identify the specific optical absorption and scattering parameters related to the firmness and pectin changes during the postharvest tissue softening;
(iii) develop efficient prediction models to determine these quality characteristics.

## 2. Materials and Methods

### 2.1. Sample Preparation

Two different peach cultivars (*Prunuspersica* L. Batsch) named ‘Baifeng’ (after blooming for 100 days) and ‘Xiahui 8’ (after blooming for 110 days) at commercial maturity were harvested from a local orchard located at 35°5′ N and 118°8′ E (Nanjing, Jiangsu, China) in June 2018 and June 2019, then transferred to the laboratory immediately for further storage experiment [19,20]. For each peach cultivar, 70 samples were selected after ensuring their uniform size and maturity and that they were without scars or insects. A total of 280 peach fruits (2 cultivars × 7 storage periods × 2 years × 10 fruits at each storage period) were prepared for further characterization. All peaches were stored at 20 °C and relative humidity (RH) > 90% for 6 days. For each cultivar, every 10 samples were randomly selected at harvest and each day for further optical and physicochemical measurements. There were two sampling points spaced at 180° intervals in every peach for optical properties and firmness measurement. The same physiochemical determinations, including the firmness and pectin compositions of each peach sample, were used. Particularly, the optical properties of peach fruit at two different wavenumber regions, the visible/short wavelength near-infrared region (600–1050 nm) and the short/medium wavelength near-infrared region (1100–1650 nm), were characterized in June 2018 and June 2019, respectively.

### 2.2. Single Integrating Sphere System

The single integrating sphere system (SIS) coupled with inverse adding doubling (IAD) was applied to acquire the optical properties of peach samples. The schematic diagram of the SIS system was reported in our studies before [21]. A 4P-GPA-033-SL integrating sphere equipment (Ø 83.82 mm, Labsphere, North Sutton, USA) was used to determine the optical properties. The inner surface of the sphere was coated with polytetrafluoroethylene (PTFE), which has a reflectivity of 98%, allowing for even light distribution [22]. There were two ports with a diameter of 25.4 mm positioned 180° apart along the equator of the integrating sphere, which were used for the entrance and exit of light. An ASBN-W100-L high-power halogen lamp (Spectral Products, Putnam, USA) was adopted as the light source to characterize the optical peripeties of peach flesh, with a maximum output power of 100 W. Furthermore, an optical fiber with a diameter of 1000 μm was used to transfer the optical signals. The optical scattering and absorption spectrum were collected based on two different spectrometers at the spectral wavelength in visible and short infrared (600–1050 nm) and near-infrared (1100–1600 nm) regions, respectively.

### 2.3. System Calibration

To ensure the SIS system’s accuracy and robustness, both distilled water and a 1% Intralipid standard solution (Sigma Aldrich, St. Louis, MO, USA) were employed. Intralipid functions as a reliable emulsion, which makes it a suitable scattering agent, whereas distilled water serves as an absorbing medium that does not exhibit scattered light. Further information about the validation techniques and results can be found in our previously published research [16].

During the calibration phase, each sample slice of peach was positioned between two quartz glass slides (1 mm thick, refractive index = 1.53) and securely held in place using a sample holder. Subsequently, the diffuse transmittance and diffuse reflectance were measured through the transmission and reflection modes of the SIS, respectively, following the previously outlined method [16]. The formulas for calculating diffuse reflectance (*Rt*) and diffuse transmittance (*Tt*) for each peach sample slice are presented in Equations (1) and (2).

(1) $$Tt=\frac{Ts-Td}{Tr-Td}$$

(2) $$Rt=\frac{Rs-Rd}{Rr-Rd}$$

where *Rd* is the dark noise reflected value of the sample; *Rr* is the reflected intensity of the reference spectrum; *Rs* is the reflected value of the sample spectrum; *Rt* is the calibrated reflectance of the sample. *Td*, *Tr*, *Ts*, and *Tt* are the spectra-measured values in the transmittance mode of the four above-mentioned conditions.

In version 3.9.10 of the IAD, each peach slice was precisely measured using a digital vernier caliper to aid in the determination of its optical properties [14]. To minimize the impact of ambient light interference, all measurements were performed in a light-proof enclosure.

### 2.4. Measurement of Optical Properties

After removing approximately 5 mm thick caps from the sample surface using a slice cutter, a pulp slice with the size of approximately 40 mm × 30 mm × 2.5 mm (length × width × thickness) was obtained for further optical property characterization [23]. The measurement methods of the optical properties and the calculation of tissue reflection and transmission were described in the aforementioned Part 2.3. All these spectral data were collected at two different spectral regions, including 600–1050 nm and 1100–1650 nm. The obtained tissue reflection and transmission spectra were substituted into IAD version 3.9.10 to calculate the absorption coefficient (*μ_a_*) and reduced scattering coefficient (*μ*′*_s_*). For each sample measurement position, three optical acquisition replicates were performed. Therefore, 840 absorption spectra (2 cultivars × 7 storage periods × 2 years × 10 fruits at each storage period × 3 optical measurement replicates) and 840 scattering spectra (2 cultivars × 7 storage periods × 2 years × 10 fruits at each storage period × 3 optical measurement replicates) were obtained in this work. 

### 2.5. Firmness Characterization

The firmness of peach flesh tissue was determined at three different locations, which were similar to the characterizing areas of optical properties. Particularly, the firmness of each peach slice was determined by the puncture tests using a digital TMS-Pro texture analyzer (FTC, Arlington, VA, USA) coupled with a cylindrical probe with a diameter of 6 mm. Particularly, the penetration speed was set at 2.0 mm/s, and the penetration depth of peach tissues at 8.0 mm. Three measurement replicates were perfumed, and the firmness (in Newtons) for each peach was calculated as the average of the measurements [22].

### 2.6. Measurement of Pectin Constitution

The water-soluble pectin and proto-pectin were extracted sequentially according to the previously described methods with appropriate modifications [21,24,25]. A flesh sample (1.25 g) near the point of optical property measurement was taken, ground, transferred to a 50 mL triangular bottle with 20 mL 95% ethanol, and bathed in water for half an hour. Filter papers were used to collect the precipitate from the extracted solutions. Afterward, 15 mL water was added and incubated at 50 °C for 30 min to dissolve the soluble pectin. After filtering, the filter paper was washed and precipitated with a small amount of water. The filtrate (water-soluble pectin) was transferred to a 50 mL volumetric bottle, and the volume was made up with water to 50 mL. Twenty milliliters of 0.5 mol/L concentrated sulfuric acid was added to the precipitate, and the solution was boiled for 60 min to hydrolyze insoluble pectin. After cooling, the solution was transferred to a 50 mL volumetric bottle, to which 0.5 mol/L sulfuric acid was added to constant volume, yielding a solution of proto-pectin.

One milliliter of pectin solution was added into a 20 mL graduated test tube, followed by the addition of 6 mL concentrated sulfuric acid along the wall of the test tube. After mixing, the mixture was incubated in boiling water for 20 min and then cooled to room temperature. This was followed by the addition of 0.5 mL 0.5% carbazole ethanol solution, vigorous mixing, and incubation in the dark for 2 h. An ultraviolet-visible spectrophotometer (UV1800, Shimadzu, Kyoto, Japan) was used to measure the absorbance at 530 nm, and the content of galacturonic acid was detected from the standard curve [26]. Formula (3) was used to obtain the pectin constitution of each component (unit: %). The standard curve determination coefficient of pectin was 0.9990. The total pectin constitution is the sum of water-soluble pectin and proto-pectin.

(3) $$C=\frac{MV}{V_{s}\cdot m\cdot 10^{6}}$$

where *C* represents the mass fraction of pectin (%); *M* indicates the amount of galacturonic acid derived from the standard curve (in micrograms, μg); *V* denotes the total volume of the sample extract (mL); *V_s_* refers to the volume of the liquid sample collected for the determination (mL), and *m* signifies the mass of the sample (g).

To better describe the changes in fruit flesh cell walls during storage, the proto-pectin index (*PI*) was calculated using Equation (4).

(4) $$PI=\frac{C_{1}}{C_{2}}$$

where *PI* is the protopectin index; *C*_1_ is the content of protopectin (%), and *C*_2_ is the content of water-soluble pectin (%).

Finally, the pectin contents of each peach fruit were characterized, and 280 reference data of peach pectin contents (2 cultivars × 7 storage periods × 2 years × 10 fruits at each storage period) were obtained for further correlation analysis and modeling development. 

### 2.7. Data Analysis

The variations in peach firmness and pectin constitutions were analyzed by variance analysis (*p* < 0.05). The internal relationships among the optical properties, firmness, and pectin constitutions were evaluated by Pearson correlation analysis. All these statistical analyses were conducted by IBM SPSS Statistics (Version 20.0). The partial least square regression (PLSR) models based on different featured optical properties were developed for the prediction of peach firmness, water-soluble pectin, protopectin, and *PI* using MATLAB software (Version R2010b). For the model development, the datasets related to all peaches harvested in 2018 and 2019 were divided into calibration and validation sets based on the K-S algorithm. Three-fourths of all experimental data was used for modeling calibration, and the other one-fourth of the data was used as the prediction set for modeling validation. The modeling procedures were repeated 10 times, and the averaged perdition results were obtained. The prediction performances of the different developed models were evaluated by the determination coefficient of the calibration set (*R_c_*^2^), the determination coefficient of the prediction set (*R_p_*^2^), and the root mean square error of calibration (*RMSEC*) and prediction (*RMSEP*), respectively.

## 3. Results and Discussion

### 3.1. Changes in Peach Firmness and Pectin Constitution

**Figure 1:**
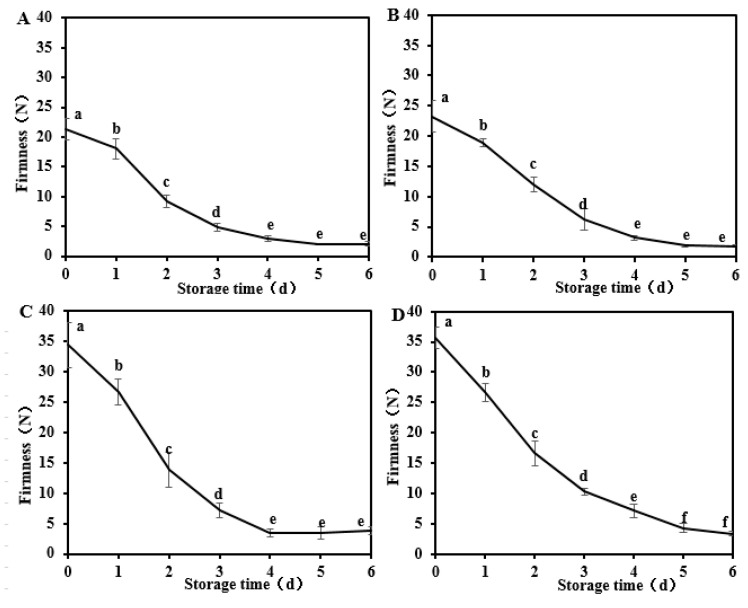
The firmness changes in ‘Baifeng’ peaches in 2018 (**A**) and 2019 (**B**) and ‘Xiahui 8’ peach flesh in 2018 (**C**) and 2019 (**D**) during the storage of 6 days at 20 ± 1 °C, respectively. Different letters within the figure (a–f) indicate significant statistical differences at the level of 0.05 (*p* < 0.05).

The firmness of ‘Baifeng’ and ‘Xiahui 8’ peaches gradually decreased during the postharvest storage of 6 days (Figure 1). Particularly, the firmness of ‘Baifeng’ peaches was significantly (*p* < 0.05) higher than that of ‘Xiahui 8’ peaches at harvest (0 d). During the storage, the firmness of ‘Baifeng’ peaches decreased by 90.3% and 92.2% in 2018 and 2019, whereas the firmness of ‘Xiahui 8’ peaches decreased by 89.0% and 90.5%, respectively. For these two cultivars, a significant decrease (*p* < 0.05) in peach tissue firmness can be observed at the primary storage of the first 4 days. Water loss and the changes in pectin structure and content were the important factors resulting in the decrease in fruit firmness. Peach is a respiratory leapfrog fruit; respiration and consumption during postharvest results in after-ripening, making the firmness softer. This result is consistent with Ma et al. [18].

**Figure 2:**
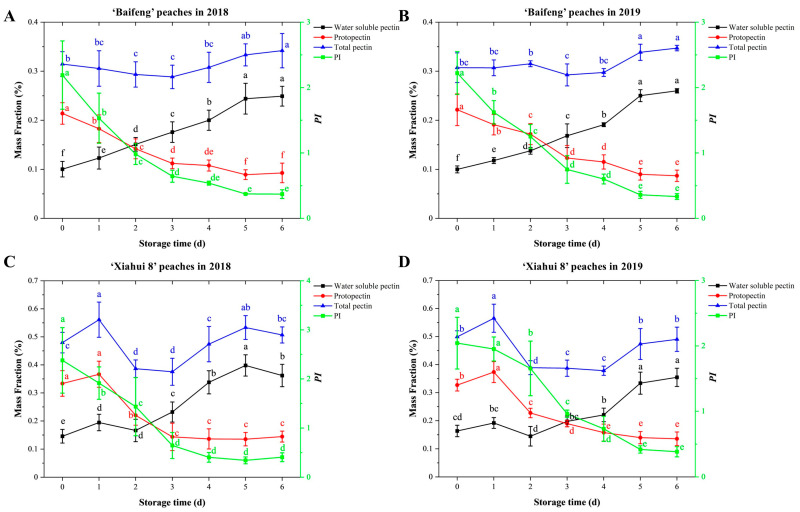
The pectin constitution changes in ‘Baifeng’ peaches in 2018 (**A**) and 2019 (**B**) and ‘Xiahui 8’ peach flesh in 2018 (**C**) and 2019 (**D**) during storage in 2018 and 2019 during the storage of 6 days at 20 ± 1 °C, respectively. Different letters within the figure indicate significant statistical differences at the level of 0.05 (*p* < 0.05).

The changes in total pectin (TP), water-soluble pectin (WSP), protopectin contents, and protopectin index (*PI*) of different peach groups during postharvest storage are shown in Figure 2. Generally, the TP content of ‘Xiahui’ peach flesh was higher than that of ‘Beifeng’ peach flesh during the postharvest storage. During the process of storing, the protopectin content and *PI* fractions in peach flesh showed a significant (*p* < 0.05) decrease, while the water-soluble pectin content gradually increased (*p* < 0.05). These pectin changes could result in the separation and dissolution of the fruit cell wall and, thus, the decrease in peach firmness during the process of tissue softening. Particularly, the *PI* index in both two peach cultivars declined sharply at the initial storage periods within 3 days, with the average content dropping from 0.51 g kg^−1^ to 0.17 g kg^−1^ after 3 days of storage, which was 66.5% lower than the harvested time (0 days). In the aspect of WSP, both two peach cultivars presented significant increases (*p* < 0.05) in WSP during the whole storage period. The increase in WSP content of peach fruit could be addressed by the solubilization of protopectin during postharvest storage [18].

### 3.2. Optical Properties

The comparison between the reference values and those derived from Mie theory indicated that the characterized values of *μ_a_* and *μ*′*_s_* in this work closely matched the previous reference values [27,28,29]. The average relative error across the range from 850 to 1650 nm was found to be 3.80%. However, experimental uncertainties resulted in baseline drift within the 600 nm to 700 nm range; for instance, light loss at the sample’s edge caused the measured values to increase [15,30]. In contrast, the fitting achieved with *μ_a_* and *μ*′*_s_* demonstrated a strong overall fit, with an average relative error of 3.43%.

#### 3.2.1. Absorption Coefficient

**Figure 3:**
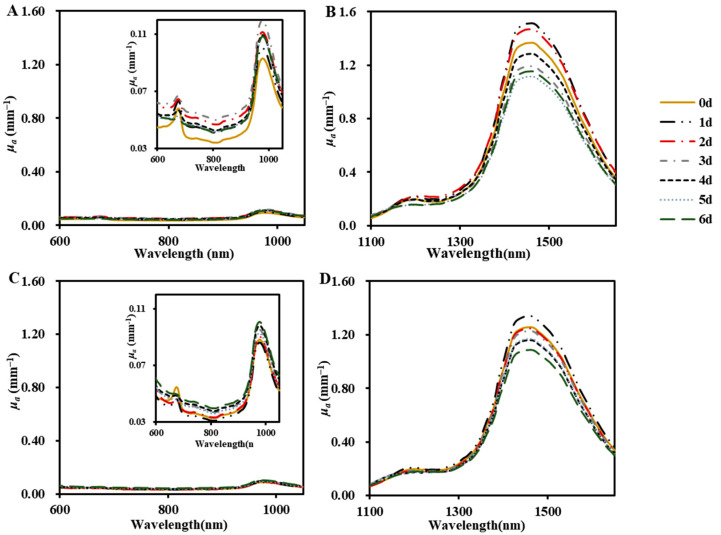
The averaged absorption coefficients (*μ_a_*) of ‘Baifeng’ (**A**,**B**) and ‘Xiahui 8’ peach flesh (**C**,**D**) at 600−1050 nm and 1100−1650 nm during the storage of 6 days, respectively.

As shown in Figure 3, the curves of the absorption coefficient *μ_a_* for ‘Baifeng’ and ‘Xiahui 8’ peaches at 600–1050 nm and 1100–1650 nm were similar at different storage periods. Four distinct absorption peaks were identified at 670 nm, 970 nm, 1170 nm, and 1460 nm, along with a relatively weaker absorption peak at 740 nm. The peak observed at 670 nm corresponded to the absorption by chlorophyll, while the peak at 740 nm was associated with the O–H stretching. According to the previous reports, the changes in moisture and pectin composition can affect fruit firmness. Water content decreased during postharvest storage, which was a signal for fruit senescence. The peaks at 970 nm and 1460 nm resulted from the overtone bands of O–H, which are linked to the molecular structure of water. Moreover, the previous results have identified the specific absorption peaks related to the changes in pectin in apples and pears. In apple cells, the depolymerization of pectin substances induces an increase in porosity (r = 0.97) and modification of cellular structure, leading to a decrease in firmness (r = 0.90). Furthermore, the firmness and cell numbers exhibited a strong positive correlation with both *μ_a_* in the range of 1000–1650 nm [22]. Additionally, the absorption peak at 1170 nm arose from the C–H stretching of macromolecular sugars [28]. Among them, the absorption peaks at 1460 nm were significantly higher than those at 670, 740, 970, and 1170 nm, with *μ_a_* values (‘Baifeng’: 1.11−1.51 mm^−1^; ‘Xiahui 8’: 1.09−1.34 mm^−1^) of more than five times higher than those of other peaks. The decreased absorption near 670 nm during storage was mainly related to the degradation of chlorophyll in two cultivar peaches, which could be consistent with the results of previous works [22,31]. It can be observed that the absorption coefficient values at 1460 nm for peach tissues initially increased and then subsequently decreased in two peach cultivars.

The average variation *μ_a_* ranges of ‘Baifeng’ and ‘Xiahui 8’ peaches in 600–1050 nm were 0.03−0.12 mm^−1^ and 0.03−0.10 mm^−1^, respectively (Figure 3A,C). This is consistent with the observations of Qin et al. [29], who observed that *μ_a_* at 500−1000 nm for peach fruit based on HISR was 0.02−0.11 mm^−1^. However, this was higher than that reported by Cen et al. [32]. The average variation ranges of *μ_a_* of ‘Baifeng’ and ‘Xiahui 8’ peaches at 1100−1650 nm was 0.05−1.51 mm^−1^ and 0.07−1.34 mm^−1^, respectively (Figure 3B,D). Although the optical properties of peach in this band have not been investigated, studies on the optical properties of apple, blueberry, and kiwifruit in this band are available [16,33]. The *μ_a_* at 1100−1650 nm of peach obtained in this experiment is within the range of the above study, confirming the reliability of these data. The optical absorption of different fruit and vegetable tissues is mainly determined by fruit and vegetable cultivar, measurement technology, and sample status.

#### 3.2.2. Reduced Scattering Coefficient

The reduced scattering coefficient (*μ’_s_*) for ‘Baifeng’ and ‘Xiahui 8’ peaches exhibited distinct troughs at wavelengths associated with significant absorption (670 nm, 740 nm, 970 nm, 1170 nm, and 1460 nm), likely due to crosstalk within the IS system. In calculating the optical property parameters using the IAD algorithm, this system tended to overestimate light absorption while neglecting light loss occurring in the integrating sphere, which resulted in elevated values of *μ_a_* and diminished values of *μ*′*_s_* [30,34]. The optical scattering in both two cultivars of peach flesh did not cohere well in the two wavebands, which did not agree with Mie law: *μ*′*_s_* decreased with the increase in wavelength. However, the law holds for both wavebands. Thus, this may be due to the difference in the performance of the spectrometer used in the two bands. In the two bands, the *μ*′*_s_* in both cultivars decreased with the increase in storage periods, which was consistent with the results of Cen et al. [35].

**Figure 4:**
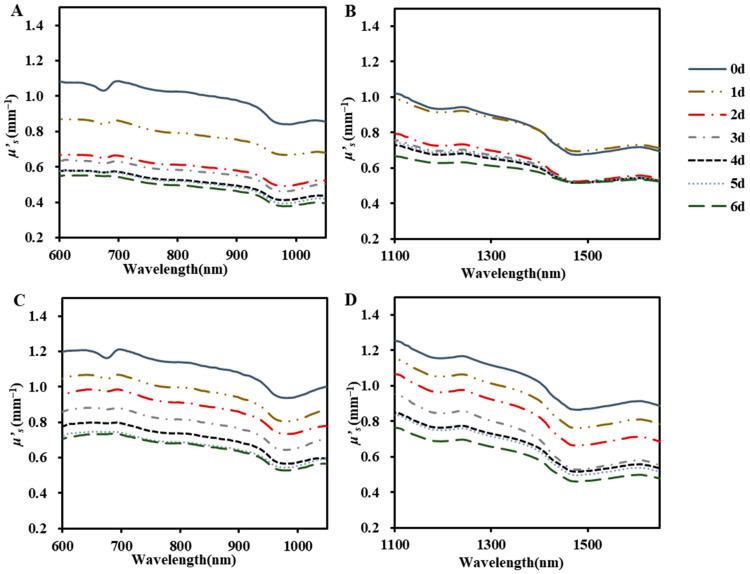
The averaged scattering coefficients (*μ*′*_s_*) of ‘Baifeng’ (**A**,**B**) and ‘Xiahui 8’ peach flesh (**C**,**D**) at 600−1050 nm and 1100−1650 nm during the storage of 6 days, respectively.

The average variation range of *μ*′*_s_* of ‘Baifeng’ and ‘Xiahui 8’ peaches at 600−1050 nm was 0.38−1.08 mm^−1^ and 0.53−1.21 mm^−1^, respectively (Figure 4A,C). In both cultivars, *μ*′*_s_* values were more than nine times higher than *μ_a_* values, which was probably because the fruits and vegetables were in a turbid medium, and the scattering property of light at 600−1050 nm was much higher than the absorption property. However, in the range of 1100−1650 nm, the optical absorption of peach tissues gradually increased, while the scattering decreased. Particularly, the *μ_a_* at 1460 nm was almost twice that of *μ*′*_s_*. At both two bands, the optical scattering of ‘Xiahui 8’ peach flesh showed relatively higher values than that of ‘Baifeng’ peach flesh, which was consistent with the higher hardness of ‘Xiahui 8’ peach flesh than that of ‘Baifeng’ peaches.

### 3.3. Relationships among Firmness, Pectin Constitution, and Optical Properties

#### 3.3.1. Relationship of Firmness with the Pectin Constitution

The results of the correlation analysis between firmness and pectin constitution of ‘Baifeng’ and ‘Xiahui’ peach tissues are shown in Table 1 and Table 2. Generally, similar correlations were observed between firmness and pectin constitution of ‘Baifeng’ and ‘Xiahui 8’ peaches in 2018 and 2019. Particularly, the firmness of both two peach cultivars was positively correlated with the protopectin content and *PI* values, whereas the firmness was negatively correlated with water-soluble pectin (WSP). In a comparison of these four pectin constitutions of both two peach cultivars, the highest correlation coefficients were observed between firmness and *PI*, with r ≥ 0.902. These results were in line with our previous results from pear and apple fruits that the increase in water-soluble pectin content in fruit tissues was mainly due to the solubilization of covalent-soluble pectin during postharvest storage. These changes in pectin resulted in the loss of fruit firmness, separation, and dissolution of cell walls. Consequently, peach firmness and their protopectin, water-soluble pectin, and *PI* content, which presented considerable internal relationships, were selected for further analysis with optical properties.

#### 3.3.2. Correlations between Absorption Coefficient, Firmness, and Pectin Constitution

**Figure 5:**
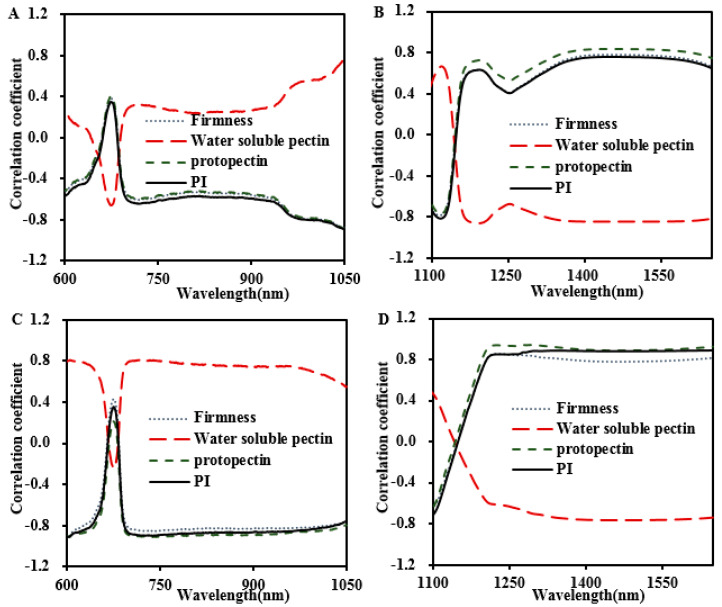
Correlations of firmness and pectin constitution with *μ_a_* at 600−1050 nm and 1100−1650 nm of ‘Baifeng’ (**A**,**B**) and ‘Xiahui 8’ peach fleshes, respectively (**C**,**D**).

In this part, the internal correlation results between the absorption coefficient (*μ_a_*), firmness of tissues, and the pectin constitutions (protopectin, water-soluble pectin, *PI*) of these two peach cultivars in 2018 and 2019 are displayed in Figure 5. At the visible wavelength range between 600 and 675 nm, the correlation coefficient (r) between *μ_a_* spectrum and tissue firmness changed rapidly from negative to positive values. At the short near-wavelength range between 675 and 1050 nm, the correlation coefficients of the absorption coefficient (*μ_a_*) and tissue firmness rapidly became negative, with the r values of −0.63 for ‘Baifeng’ and −0.83 for ‘Xiahui 8’, respectively. At the near-infrared wavelength between 1100 nm and 1209 nm, the negative correlation coefficient decays and turns positive within 1209–1650 nm, with the r values of 0.68 for ‘Baifeng’ and 0.81 for ‘Xiahui 8’ peach fruit. The range and trend of the r values between protopectin, the *PI*, and the *μ_a_* were similar to those of the *μ_a_* with firmness.

Overall, positive correlations between water-soluble pectin and the *μ_a_* were investigated, while negative r values showed at 670 nm. Within 675–1050 nm, the correlation coefficient rapidly attained a positive value and maintained a positive correlation after 700 nm, with the r values of 0.36 for ‘Baifeng’ and 0.80 for ‘Xiahui 8’. The r at 1100–1209 nm changed rapidly from positive to negative and maintained a negative correlation at 1209–1650 nm, with the r values of −0.81 for ‘Baifeng’ and −0.74 for ‘Xiahui 8’. Additionally, the correlation between the *μ_a_* and water-soluble pectin was the highest at 600–1050 nm, while the *PI* was the highest at 1100–1650 nm in ‘Baifeng’ peach. The average correlation between protopectin, the *PI*, and the *μ_a_* in the whole band range of ‘Xiahui 8’ peach was higher than that of other pectin types.

#### 3.3.3. Correlations between Reduced Scattering Coefficient, Firmness, and Pectin Constitution

**Figure 6:**
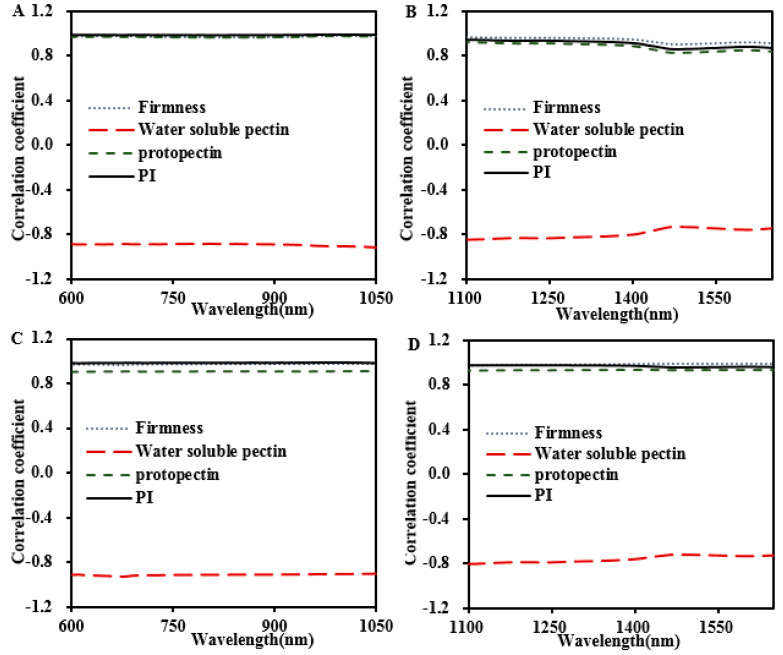
Correlations of firmness and pectin constitution with *μ’_s_* at 600−1050 nm and 1100−1650 nm of ‘Baifeng’ (**A**,**B**) and ‘Xiahui 8’ peach fleshes, respectively (**C**,**D**).

In this part, the internal correlations between the reduced scattering coefficients (*μ*′*_s_*), the tissue firmness, and the pectin constitution of two peach cultivars are presented in Figure 6. Generally, all these correlation values presented limited variations both in visible and near-infrared wavelength regions; thus, no obvious reduced scattering spectral features could be selected. Particularly, the *μ*′*_s_* values for both peach cultivars correlated positively with tissue firmness. A considerable positive correlation was found between the reduced scattering coefficient (*μ*′*_s_*) of peach flesh at 675 nm and their firmness (r values = 0.932 and 0.992, respectively). Conversely, the negative correlation between *μ*′*_s_* and firmness at 790 nm and 912 nm was reported [36]. The firmness of the fruit is influenced by cell wall degradation and water loss. Both cell wall degradation and water loss can strongly impact light propagation in fruit tissues [16,36]. Among these processes, cell wall breakdown leads to cell separation and simplifies the intracellular structure, reducing the number of scattering boundaries and consequently decreasing light dispersion. Furthermore, the cell wall breakdown may weaken the cell structure adhesion and form different intercellular refractive boundaries, thus enhancing the optical scattering properties [37,38]. Simultaneously, the water loss during the postharvest storage of peaches increased both the volume and the number of the cell wall intercellular spaces while diminishing the sizes of the cells. Air trapped in these spaces creates refractive index mismatches, which, in turn, leads to heightened light scattering. Notably, *μ’_s_* showed a positive correlation with protopectin and *PI* but a negative correlation with water-soluble pectin.

Furthermore, *PI* showed the highest correlation with *μ*′*_s_*, with r ≥ 0.973. The relationship between pectin constitution and optical properties is in line with the results mentioned in Section 3.3.1, confirming that *PI* may be an important factor affecting the firmness–optical property correlations. Additionally, the mean correlation coefficient of firmness, pectin constitution, and *μ*′*_s_* was higher than that of *μ_a_* in both cultivars, which may be because pectin constitution in the cell wall affects microstructural changes, causing changes in optical scattering [22]. 

The relationship of *μ*′*_s_* with the firmness and *PI* at a fixed wavelength was studied next. Finally, linear fitting equations of firmness, *PI*, and *μ*′*_s_* were established at wavelengths of 660 nm, 950 nm, 1203 nm, and 1453 nm. As shown in Table 3, the determination coefficient *R*^2^ of the linear fitting equation of firmness, *PI*, and *μ*′*_s_* in ‘Baifeng’ peaches was ≥0.764, while it was ≥0.926 for ‘Xiahui 8’ peaches.

### 3.4. Prediction of Peach Firmness and Pectin Constitution Based on Optical Property

Based on the aforementioned correlations between the optical properties and peach quality parameters, the partial least square regression (PLSR) models were established based on the *μ_a_* and *μ*′*_s_* coefficients at 600–1050 nm and 1100–1650 nm, to evaluate the prediction ability for peach firmness, water-soluble pectin, proto-pectin, and *PI* contents (Table 4). The best prediction performances of the developed PLS model were obtained for the firmness of these two cultivars, with the *R_p_*^2^ values of 0.863 and the *RMSEC* values of less than 4.253 N. Particularly, the PLS models based on the optical scattering properties at near-infrared region (1100–1650 nm) can provide better prediction of peach firmness than that of visible and short near-infrared spectral ranges (600–1050 nm). These prediction results were better than the previously reported prediction models for apple and pear firmness based on optical properties [34,36,38]. Moreover, the PLS models based on *μ*′*_s_* coefficients showed relatively better prediction results than the models using *μ_a_* coefficients for peach firmness, pectin constitution, and optical properties of both two cultivars. This result further proves that the correlations between firmness, pectin constitution, and scattering properties were better than those of absorption properties. In addition, in both cultivars, the prediction model of *PI* was optimal, and the *R_p_*^2^ was 0.751−0.812. Consistent with the conclusion in Section 3.2.2, *PI* contents in peach tissues showed the highest correlation with optical scattering. This indicates that the strong correlations between optical scattering characteristics and *PI* could facilitate the prediction of firmness through optical technology that utilizes visible/short-wavelength near-infrared and short/medium-wavelength near-infrared regions. The uses of optical scattering properties have the possibility to evaluate both the peach firmness and their internal variations in pectin constitution.

## 4. Conclusions

This study investigated the effect of pectin constitution, including total pectin, water-soluble pectin, protopectin contents, and protopectin index (*PI*), on the optical properties and firmness of ‘Baifeng’ and ‘Xiahui 8’ peach flesh at the different softening degree during postharvest storage. The firmness of both cultivars of peaches correlated well with *PI*. In addition, the firmness and *PI* correlated well with optical scattering, and the *μ*′*_s_* at 660 nm, 950 nm, 1203 nm, and 1453 nm were identified. Furthermore, the PLSR prediction model based on *μ*′*_s_* had the potential to evaluate the firmness and pectin constitutions of peach flesh. The prediction models based on *μ*′*_s_* are more suited to describe the firmness and pectin changes in peach fruit than those based on *μ_a_* values. Our results indicated that the high correlations between optical scattering properties and *PI* might explain the firmness prediction using optical technology based on visible/short wavelength near-infrared and short/medium wavelength near-infrared regions. Scattering properties may be potentially used to evaluate the pectin constitution in peaches. Future works could develop rapid and effective optical sensors for the online determination of peach firmness based on the combination of optical scattering and imaging techniques, in particular, with the spatially resolved spectroscopy and spatially resolved hyperspectral diffuse reflectance imaging technique.

## Figures and Tables

**Table 1:** Correlation analysis of firmness and pectin constitution during the storage of ‘Baifeng’ peach flesh.

	Firmness	Total Pectin	Water-Soluble Pectin	Proto-Pectin	*PI*
Firmness	1	−0.268	−0.895 **	0.901 **	0.948 **
Total pectin		1	0.587 *	−0.269	−0.333
Water-soluble pectin			1	−0.876 *	−0.897 **
Proto-pectin				1	0.971 **
*PI*					1

* *p* < 0.05, ** *p* < 0.01.

**Table 2:** Correlation analysis of firmness and pectin constitution during the storage of ‘Xiahui 8’ peach flesh.

	Firmness	Total Pectin	Water-Soluble Pectin	Proto-Pectin	*PI*
Firmness	1	0.347	−0.681 *	0.903 *	0.912 **
Total pectin		1	0.343	0.545	0.100
Water-soluble pectin			1	0.601 *	−0.816 **
Proto-pectin				1	0.897 **
*PI*					1

* *p* < 0.05, ** *p* < 0.01.

**Table 3:** Linear fitting equations of firmness, *PI*, and the reduced scattering coefficients (*μ’_s_*) at 660 nm, 950 nm, 1203 nm, and 1453 nm for ‘Baifeng’ and ‘Xiahui 8’ peaches during the postharvest storage of 6 days in 2018 and 2019.

	Parameters	Wavelength (nm)	Equation	*R* ^2^
Baifeng	Firmness	660	*y* = 0.022*x* + 0.499	0.950
		950	*y* = 0.0216*x* + 0.369	0.946
		1203	*y* = 0.0134*x* + 0.623	0.917
		1453	*y =* 0.0089*x* + 0.497	0.830
	*PI*	660	*y* = 0.262*x* + 0.442	0.982
		950	*y* = 0.257*x* + 0.312	0.922
		1203	*y* = 0.160*x* + 0.588	0.876
		1453	*y =* 0.104*x* − 0.477	0.764
Xiahui 8	Firmness	660	*y* = 0.013*x* + 0.737	0.935
		950	*y* = 0.012*x* + 0.561	0.951
		1203	*y* = 0.014*x* + 0.681	0.969
		1453	*y =* 0.0126*x* + 0.450	0.987
	*PI*	660	*y* = 0.201*x* + 0.695	0.964
		950	*y* = 0.186*x* + 0.523	0.970
		1203	*y* = 0.237*x* + 0.61	0.956
		1453	*y =* 0.211*x* + 0.392	0.926

Abbreviation: *μ*′*_s_* represents the reduced scattering coefficient (mm^−1^); *R*^2^ indicates the determination coefficient.

**Table 4:** The prediction performances of PLS models for the firmness and pectin constitution of ‘Baifeng’ and ‘Xiahui 8’ peach flesh in 2018 and 2019, based on optical properties at 600−1050 nm and 1100–1650 nm, respectively.

	Wavelength	Parameters	Optical Property	Calibration		Validation	
	(nm)			*R_c_* ^2^	*RMSEC*	*R_p_* ^2^	*RMSEP*
Baifeng	600–1050	Firmness	*μ_a_*	0.774	3.594	0.766	3.817
			*μ*′*_s_*	0.802	2.820	0.850	2.930
		Water-soluble pectin	*μ_a_*	0.821	0.024	0.779	0.026
			*μ*′*_s_*	0.797	0.026	0.795	0.027
		Protopectin	*μ_a_*	0.745	0.024	0.715	0.026
			*μ* *’_s_*	0.803	0.021	0.765	0.022
		*PI*	*μ_a_*	0.653	0.421	0.650	0.366
			*μ*′*_s_*	0.852	0.268	0.811	0.290
	1100–1650	Firmness	*μ_a_*	0.680	4.608	0.668	4.593
			*μ*′*_s_*	0.856	3.093	0.854	3.085
		Water-soluble pectin	*μ_a_*	0.712	0.032	0.702	0.042
			*μ*′*_s_*	0.670	0.034	0.642	0.033
		Protopectin	*μ_a_*	0.750	0.025	0.705	0.050
			*μ* *’_s_*	0.752	0.026	0.738	0.028
		*PI*	*μ_a_*	0.721	0.361	0.690	0.357
			*μ*′*_s_*	0.816	0.293	0.812	0.319
Xiahui 8	600–1050	Firmness	*μ_a_*	0.765	5.79	0.761	5.888
			*μ*′*_s_*	0.848	4.7	0.845	4.436
		Water-soluble pectin	*μ_a_*	0.710	0.055	0.687	0.056
			*μ*′*_s_*	0.751	0.048	0.704	0.064
		Protopectin	*μ_a_*	0.608	0.065	0.590	0.030
			*μ*′*_s_*	0.723	0.053	0.714	0.051
		*PI*	*μ_a_*	0.691	0.453	0.691	0.565
			*μ*′*_s_*	0.758	0.413	0.751	0.449
	1100–1650	Firmness	*μ_a_*	0.684	5.456	0.668	8.248
			*μ*′*_s_*	0.870	4.076	0.863	4.253
		Water-soluble pectin	*μ_a_*	0.630	0.050	0.615	0.048
			*μ*′*_s_*	0.634	0.049	0.630	0.057
		Protopectin	*μ_a_*	0.740	0.047	0.712	0.038
			*μ*′*_s_*	0.796	0.042	0.778	0.057
		*PI*	*μ_a_*	0.738	0.360	0.709	0.362
			*μ*′*_s_*	0.835	0.299	0.802	0.403

Abbreviation: *R_c_*^2^ represents the determination coefficient of calibration; *R_p_*^2^ indicates the determination coefficient of validation; *RMSEC* means the root mean square error of calibration; RMSEP denotes the root mean square error of validation.

## Data Availability

The original contributions presented in the study are included in the article, further inquiries can be directed to the corresponding author.
